# Plant Tissues in 3D *via* X-Ray Tomography: Simple Contrasting Methods Allow High Resolution Imaging

**DOI:** 10.1371/journal.pone.0075295

**Published:** 2013-09-27

**Authors:** Yannick M. Staedler, David Masson, Jürg Schönenberger

**Affiliations:** 1 Department of Structural and Functional Botany, University of Vienna, Vienna, Austria; 2 Federal Office of Meteorology and Climatology, MeteoSwiss, Zurich, Switzerland; Wuhan University, China

## Abstract

Computed tomography remains strongly underused in plant sciences despite its high potential in delivering detailed 3D phenotypical information because of the low X-ray absorption of most plant tissues. Existing protocols to study soft tissues display poor performance, especially when compared to those used on animals. More efficient protocols to study plant material are therefore needed. Flowers of *Arabidopsis thaliana* and *Marcgravia caudata* were immersed in a selection of contrasting agents used to treat samples for transmission electron microscopy. Grayscale values for floral tissues and background were measured as a function of time. Contrast was quantified *via* a contrast index. The thick buds of *Marcgravia* were scanned to determine which contrasting agents best penetrate thick tissues. The highest contrast increase with cytoplasm-rich tissues was obtained with phosphotungstate, whereas osmium tetroxide and bismuth tatrate displayed the highest contrast increase with vacuolated tissues. Phosphotungstate also displayed the best sample penetration. Furthermore, infiltration with phosphotungstate allowed imaging of all plants parts at a high resolution of 3 µm, which approaches the maximum resolution of our equipment: 1.5 µm. The high affinity of phosphotungstate for vasculature, cytoplasm-rich tissue, and pollen causes these tissues to absorb more X-rays than the surrounding tissues, which, in turn, makes these tissues appear brighter on the scan data. Tissues with different brightness can then be virtually dissected from each other by selecting the bracket of grayscale to be visualized. Promising directions for the future include *in silico* phenotyping and developmental studies of plant inner parts (*e.g.*, ovules, vasculature, pollen, and cell nuclei) *via* virtual dissection as well as correlations of quantitative phenotypes with omics datasets. Therefore, this work represents a crucial improvement of previous methods, allowing new directions of research to be undertaken in areas ranging from morphology to systems biology.

## Introduction

The phenotype, *i.e.* the total sum of the observable characteristics of an organism, needs to be quantified both with precision and detail in order to harvest the full potentialities of the omics era. The most complete way to describe the morphological phenotype of a given object is to build a three dimensional (3D) model. The immense amount of data contained in an accurate 3D model can then be mined in order to explore correlations with other types of data, *e.g.* metabolite content [1], pollinator [2], or crop yield [3].

Reconstruction of the 3D structure of a sample can either be done *via* two-dimensional sections or *via* whole mount studies. Reconstructing an object *via* sections is highly facilitated by existing semi-automatic methods to align the sections [4]. However, the work-load is still high, and destruction of the samples is required. Whole mount methods include Micro Magnetic Resonnance Imaging (microMRI), Optical Projection Tomography (OPT), and High Resolution X-ray Computed Tomography (HRXCT). MicroMRI requires large and very expensive magnets and cannot currently achieve resolution below 50–25 µm [5], [6], [7]. OPT requires that samples be transparent, which implies that clearing with sodium hydroxide (caustic soda) is needed before samples can be visualized. The resolution of OPT is *ca.* 15–10 µm [8] and sample size is typically a few mm [9]. In contrast, HRXCT requires minimal/no sample preparation and is not destructive. Furthermore, HRXCT can be performed on specimens whose sizes lie in the meter range. HRXCT can be performed either with: (1) synchrotron radiation, which delivers high X-Ray fluxes, allowing short scanning times, but with limited, competitive availability, or (2) commercially available X-Ray sources, which deliver low fluxes, leading to long scanning times, but with ready availability, because included in commercial scanners. The maximal resolution that can be currently obtained with HRXCT is 30 nm with synchrotron radiation, and 50 nm with commercially available sources.

Although originally developed for medical diagnosis in the early 1970s [10], [11], [12], computed tomography found use in a wide range of fields from oncology to material sciences (see Table S1). In plant sciences, HRXCT was first used to study root development [13], [14], [15], [16], [17], [18], [19]. It was later used to study fossils [20], [21], [22], [23], [24] and plant features that segregate from their background due to strong differences in density or that comprise dense tissue themselves such as seeds (void network structure) [25], cellular structure [26]), calcium oxalate crystals [27], graft structure [28], leaf structure in resurrection plants [29], [30], and vasculature [31], [32]. However, no studies were carried out on soft tissues such as young organs, meristems, and flowers, with the notable exception of van der Niet et al. 2010 [2]. The latter study was performed at low resolution and focussed on flower surface only.

In plant sciences as a whole, studies making use of computed tomography have remained comparatively scarce. A search on the ISI Web of Knowledge^SM^, with the topic “computed tomography”, was refined per “Subject Area”, over the last decade (Table S1). The part of articles in the Subject Area “plant sciences” that comprises the topic “computed tomography” is *ca*. 6x less than in “zoology”, and 27x less than in an average ISI-referenced publication. Moreover, the proportion of the papers containing the topic “computed tomography” in plant sciences is growing much slower than in other Subject Areas (Fig. S1). Thus, computed tomography is comparatively underused in plant sciences, and there is little meaningful trend towards change.

The reason for the underuse of HRXCT in plant sciences is that plants tissues are mostly constituted of light elements, which display low X-ray absorptions. HRXCT relies on the partial absorption of X-Rays by the sample (for our equipment 65–80%); therefore, very low-energy X-Rays have to be used for proper imaging. Under such conditions, typical X-ray sources have low yields, therefore requiring very long scanning times. Long scanning times not only decrease throughput but also decrease scan quality by increasing the probability of motion artefacts. These problems are especially acute in the case of very young and meristematic tissues without well-differentiated cell walls.

An HRXCT scan consists of a large number of pictures of X-ray shadows of an object, typically *ca.* 1000, each carried out at a different rotation angle. These pictures are then processed by algorithms that reconstruct a volume model of the sample [33]. However, whatever is contained in the field of view around the sample will also be reconstructed. The differentiation between sample and background is typically carried out using methods that rely on grayscale differences, which are linked to differences in X-ray absorption. Moreover, strong differences of X-ray absorption between the sample and the background decrease the workload of the investigator. As previously mentioned, plants are mostly composed of weakly absorbing elements. Therefore, plant samples scanned in alcohol or water will display neither strong contrast between sample and background nor between different tissues within the sample.

The above-mentioned problems can be solved by infiltrating the samples with heavy metals, since X-ray absorption increases with atomic number. Protocols were already described to increase the X-ray absorption of animal [34], [35] and plant tissues [36], [37]. However, in order obtain high resolution and high magnification imaging, the protocols for plant tissues involve critical point drying of the samples [36], [37] and infiltration with iodine [36] or toxic uranium acetate [37]. Furthermore, these protocols [36], [37] do not include quantification of contrast increase nor proved satisfactory when replicated, because they caused only weak contrast increase and displayed poor tissue penetration. Therefore, the TEM literature on sample preparation [38], in which low sample absorptions are also solved by heavy metal infiltration, was searched for more efficient and straightforward full-mount protocols. Nine compounds were selected and were tested in 13 protocols. Mounting techniques adapted to sample size were also developed, and special protocols were established to reach the resolution limit of our equipment (1.5 µm), even for the most difficult samples (very young, very small, low density, meristematic samples).

For each infiltration protocol, the following properties were assessed: (1) contrast improvement, (2) repeatability and uniformity of the contrasting agent, and (3) selectivity of contrasting agent. Penetration of the tissues was qualitatively assessed.

## Materials and Methods

### Materials

Anthetic flowers of *Arabidospsis thaliana* (wt ecotype Col-0, and double line *ap1 cal pAP1::AP1-GR*) were used as a study object because they are small, easy to obtain, very well-described, and comprise both complex organs and a wide range of tissues. Furthermore, *A. thaliana* is by far the most favoured plant model. Late buds of *Marcgravia caudata* (Marcgraviaceae; 2.5–3.5 mm diameter) were scanned in order to qualitatively assess the penetration power of the contrasting agent through thick and dense tissue barriers (see below). In order to demonstrate the versatility of our methods, larger and more complex flowers were also scanned from two other species: *Calycanthus floridus* (Calycanthaceae), and *Haplophyllum lissonotum* (Rutaceae).

*Arabidopsis thaliana* samples (anthetic wild type flowers and 6d old flower meristems of double line *ap1 cal pAP1::AP1-GR* mutant with inducible, synchronous flower development; courtesy Toshiro Ito research group, Temasek Life Sciences Laboratory, National University of Singapore, unpublished data) and buds of *Marcgravia caudata* were fixed in FAA (50 vol ethanol 100%, 5 vol glacial acetic acid, 10 vol formaldehyde 37%, 35 vol dH2O) and washed with 70% ethanol (EtOH) prior to infiltration. All other plants samples were fixed and preserved in 70% EtOH.

Flowers were infiltrated for ½, 1, 2, and 8 days. For each infiltration protocol, five flowers were scanned for each infiltration time (260 total). The investigated contrasting agents are summarized in Table 1.

**Table 1:** Infiltration solutions tested for contrast increase.

Element	Compound	Concentration	Solvent	Reference	Abbreviaton
Mn	Potassium permanganate	1% (w/vol)	dH2O	[38]	Mn
Fe	high Iron diamine	69 mM	dH2O	[38]	Fe
Ru	ruthenium red	0.02% (w/vol)	dH2O	[38]	Ru
I	Lugoĺs solution	13 mM I2+41 mM KI	dH2O	Merk Chemicals	I.lugol
I	EtOH iodine	1% (w/vol)	Ethanol 96%	[35]	I.EtOH
I	Aqueous Iodine	1% I_2_+2% KI (w/vol)	dH2O	[35]	I.H2O
W	phosphotungstate	1% (w/vol)	Ethanol 96%	[38]	W/EtOH
W	phosphotungstate	1% (w/vol)	FAA	original	W/FAA
Os	osmium tetroxide	1% (w/vol)	dH2O	[38]	OsO4
Os	osmium tetroxide + ferrocyanate	0.5% OsO_4_+0.4% Fe(CN)_6_	dH2O	[38]	OsFeCN
Pb	lead citrate	3% (w/vol)	dH2O	[38]	Pb
Bi	bismuth tartrate	68 mM	H2O (2N NaOH)	[38]	Bi

### Mounting

**Figure 1:**
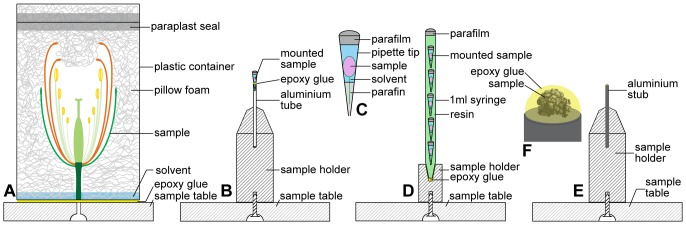
Sample mounting techniques for samples of different sizes. (A) Mounting of large samples (>10 mm). Samples are mounted in acryl foam and scanned in a solvent atmosphere, which provides optimal signal to background ratio. (B–D) Mounting of medium-sized samples (1–10 mm). For medium-sized samples, movements of solvent surface cause significant sample movement; therefore samples are best scanned immersed in the solvent. (B) Single sample are mounted in pipette tips, which are inserted in aluminium tubes and glued to the latter. (C) In the pipette tips, paraffin wax is used as a seal and to stabilize the sample on the lower end of the tip. PARAFILM is used to seal the upper end of the tip. (D) Sample batch with batch holder. Batch scanning is required to scan large numbers of samples. Samples mounted in pipette tips are mounted in a 1 ml syringe tube and stabilized with resin. (E, F) Mounting of small samples (>1 mm). (E) Small sample with sample holder. (F) Small samples require maximal stabilization and contrast enhancement. Such samples are best scanned after having been critical point dried and embedded in a drop of epoxy glue directly on aluminium stubs.

After infiltration, *Arabidopsis thaliana* flowers (medium sized samples with diameter 1–10 mm) were washed three times with 1 ml 70% EtOH, and were mounted individually in 250 µl pipette tips (Semadeni, polypropylene). Inside the pipette tips, the samples were submersed in 70% EtOH. On the lower, narrow end of the tip, parafine wax (HISTOSEC®, Merck) was used to stabilize the samples by embedding the flower stem in the wax, and to seal the lower end of the pipette tip. The upper, broader end of the tip was cut off at *ca.* 7 mm above the sample. In order to keep the samples immersed in 70% EtOH during storage and scanning, an additional 5 mm of 70% EtOH were added in the tip as a reserve. PARAFILM (©Pechiney) was used to seal the upper end of the pipette tip (Fig. 1C). Samples were either scanned singly or in batches of five. Single samples were mounted in pipette tips (see above), and were then glued to an aluminium tube, which was itself held by a sample holder (Fig. 1B). When mounted in batches, the samples were first mounted in pipette tips like single samples and were then stacked in 1 ml syringe tubes (CODAN Medical ApS), five samples per tube. The syringe tubes were then filled with polyester resin (GT type, Kurt Wolf & Co KG) to prevent movement (Fig. 1D). The 1 ml syringe tubes containing batches of five samples were glued (UHU PLUS®, UHU GmbH & Co) on an in–house aluminium holder.

Large samples (>10 mm diameter) were mounted in 50–200 ml cylindrical containers (VWR international, polypropylene), stabilized with acryl pillow foam (DACRON®COMFOREL® INVISTA Ltd.). After mounting, two millilitre 70% EtOH were poured along the vial wall, in order to keep the samples in an ethanol atmosphere. The containers were sealed with PARAFILM (Fig. 1A). The samples were left to settle 6–8 h prior to scanning, because freshly mounted samples slightly change their positions. The containers were glued to the sample table (XRadia Inc.) with epoxy glue (UHU PLUS®).

Small samples (>1 mm) were critical point dried (autosamdri®-815, tousimis res. corp.) and sputtered with gold for three minutes (SCD050, BALZERS AG). They were mounted in a drop of epoxy glue (UHU PLUS®), directly on aluminium stubs (Fig. 1E, 1F). The contrast between sample and air (or glue) is higher than between sample and EtOH, thereby allowing a better imaging of small features.

### Scanning

The scans were performed on an XRadia MicroXCT-200 system. X-Ray detection was performed *via* scintillator crystals (XRadia Inc. in-house) that convert X-rays into visible light. The light was then focused (Nikkon optical lense) and detected *via* a CCD camera. The X-ray source was a Hammamatsu L9421-02 90 kV Microfocus X-Ray source. Scanning conditions are detailed in table 2. Dynamic ring removal (small translations associated with sample rotation) was used in order to prevent the formation of ring artefacts in the 3D model. The scanning conditions were optimized for fast scanning and practicality. One batch of five *A. thaliana* samples was scanned in 11h30, therefore allowing two batches to be scanned daily.

**Table 2:** Material, treatment and scanning parameters.

Species	Accessionnumber	Materialtype	Fixation	Contrastingagent	Immersion time [d]	CP drying	Acceleration voltage [kV]	Source current[µA]	Exposure time [s]	Picturespersample	Camera binning	Optical magn.	Pixelsize[µm]	Illustration/purpose
*Arabidopsis thaliana*	NA	O pen flower	FAA	see list table 1	0.5–8	no	25	100	7	728	2	10x	1.5	Grayscale measurements
*Marcgravia caudata*	JS 823	late bud	FAA	none	-	no	35	200	5	1000	2	1x	18.5	Fig. 4
*Marcgravia caudata*	JS 823	late bud	FAA	bismuth tartrate	8	no	35	200	5	1000	2	1x	18.5	Fig. 4
*Marcgravia caudata*	JS 823	late bud	FAA	potassium permanganate	8	no	35	200	5	1000	2	1x	18.5	Fig. 4
*Marcgravia caudata*	JS 823	late bud	FAA	phosphotungstate	8	no	35	200	5	1000	2	1x	18.5	Fig. 4
*Marcgravia caudata*	JS 823	late bud	FAA	Lugoĺs solution	8	no	35	200	5	1000	2	1x	18.5	Fig. 4
*Marcgravia caudata*	JS 823	late bud	FAA	lead citrate	8	no	35	200	5	1000	2	1x	18.5	Fig. 4
*Marcgravia caudata*	JS 823	late bud	FAA	osmium tetroxide	8	no	35	200	5	1000	2	1x	18.5	Fig. 4
*Marcgravia caudata*	JS 823	late bud	FAA	uranium acetate	8	no	35	200	5	1000	2	1x	18.5	Fig. 4
*Arabidopsis thaliana*(wt ecotype Col-0)	NA	open flower	FAA	potassium permanganate	2	no	25	100	7	728	2	10x	1.5	Fig. 5A
*Arabidopsis thaliana*(wt ecotype Col-0)	NA	open flower	FAA	osmium tetroxide	8	no	25	100	7	728	2	10x	1.5	Fig. 5B
*Arabidopsis thaliana*(wt ecotype Col-0)	NA	open flower	FAA	lead citrate	8	no	25	100	7	728	2	10x	1.5	Fig. 5C
*Arabidopsis thaliana*(wt ecotype Col-0)	NA	open flower	FAA	phosphotungstate	8	no	25	100	7	728	2	10x	1.5	Fig. 5D
*Arabidopsis thaliana*(wt ecotype Col-0)	NA	open flower	FAA	bismuth tartrate	2	no	25	100	7	728	2	10x	1.5	Fig. 5E
*Arabidopsis thaliana*(wt ecotype Col-0)	NA	open flower	FAA	potassium permanganate	2	no	25	100	7	728	2	10x	1.5	Fig. 5F
*Calycanthus floridus*	YS04–87	anthetic carpel	70%EtOH	bismuth tartrate	8	no	30	200	10	1000	1	20x	1.1	Fig. 5G
*Haplophyllum* *lissonotum*	SM 40	open flower	70%EtOH	phosphotungstate	8	no	40	200	10	1500	1	4x	2.75	Fig. 5H
*Arabidopsis thaliana*(wt ecotype Col-0)	NA	open flower	FAA	phosphotungstate	8	no	25	100	28	1600	1	10x	0.97	Fig. 6A–D
*Arabidopsis thaliana*(double line ap1cal pAP1::AP1-GR )	NA	youngdeveloppingflowers	FAA	phosphotungstate	8	yes	40	100	30	1600	1	20x	0.45	Fig. 7A–B, Video S1, S2
*Arabidopsis thaliana*(wt ecotype Col-0)	NA	gynoecium fromopen flower	FAA	phosphotungstate	8	no	25	100	25	1600	1	20x	0.5	Fig. S4, Video S3, S4

### 3D Reconstruction

XMReconstructor 8.1.6599 (XRadia Inc.) was used to perform the 3D reconstruction from the scanning data. The AMIRA-based XM3DViewer 1.1.6 (XRadia Inc.) was used to visualize the scans. The reconstructed 3D data were then exported with XMController 8.1.6599 to series of pictures (tiff format) of reconstructed sections through the sample, typically *ca.* one thousand per sample.

### Grayscale Measurements

The abovementioned series of pictures were imported into the MIMICS Innovation Suite 14.1 (Materialise NV), in order to measure the grayscale values of the different organs. Grayscale values were measured for the following floral parts: sepals, petals, filaments, pollen grains, ovary wall, ovules (integuments), and stigmatic papillae. Each measurement was repeated three times on each tissue, on each sample. Measurements of the grayscale value of the same tissue within the same sample were performed on different reconstructed sections. Grayscale values were measured on circular areas of 50 µm^2^ for the tissues and 200 µm^2^ for the solvent.

### Contrast Index

The measured grayscale values of a tissue “Gsc(tissue)” were transformed into a Weber contrast index (CI) [39], in order to quantify contrast, and correct for scaling and additive factors:
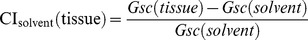
(1)


CI values were then used to quantify contrast improvement.

### Penetration of the Contrasting Agent: *Arabidopsis* (Small Sample)

It was assumed that the concentration of the contrasting agent in the tissues followed a simple exponential saturation curve.
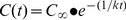
(2)


With C(t) = concentration at time t; C_∞_ = concentration after infinitely long infiltration time; k = constant; t = time. In order to assess and compare the penetration powers of the different contrasting agents, the time that contrasting agents required to reach half-saturation (t_1/2_) was calculated. It was assumed that 90% saturation was attained after 8d infiltration (C_8d_ = 0.9 C_∞_). The half-saturation time is therefore:
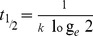
(3)


In order to obtain a measure of infiltration speed and the reliability of this speed, equation (2) was regressed against a randomized selection of one point per infiltration time (1/2 day, 1 day, 2 days, and 8 days), per tissue, and per contrasting agent (see table 1). This procedure was repeated 1000 times. Therefore, for each combination of tissue and contrasting agent, 1000 exponential saturation relations were obtained. Equation (3) was then used to calculate 1000 half-saturation times (t_1/2_) from these relations. The distribution of the 1000 half-saturation times was then plotted, for each contrasting agent, for each tissue. Median and interquartile range of the half-saturation time distributions were used as proxies for infiltration speed and reliability respectively.

### Penetration of the Contrasting Agent: *Marcgravia Caudata* (Large Sample)

*Arabidospsis thaliana* flowers are open (easily accessible to chemicals) and very small, with organs only a few cell layers thick. Therefore, contrasting agents do not need to diffuse across large distances to reach all the organs of the flower. *M. caudata* buds possess a thick and hermetic protection layer made of fused petals. Thus, in order to test diffusion of the contrasting agents into tightly closed samples with many cell layers, *M. caudata* buds were infiltrated with a subset of the contrasting agents in table 1 for one week. The samples were mounted in acryl foam and scanned (see table 2 for scan settings). Transverse sections through the buds were reconstructed at the level where both the style and the pollen sacks are present. The grayscale values of the tissues of the bud were measured along a continuous line from one side of the bud to the other, across the fused petals, stamens, and style, thereby yielding a grayscale profile across the bud. The grayscale profiles were then graphically converted into contrast index profiles by moving the x-axis (the values of the background were set to zero), and by scaling the graph along the y-axis to match calculated values. Contrast index profiles allowed quantitative comparison of contrast increase among the flower organs of the same bud, and between contrasting agents on different buds. The samples were scanned in a solvent saturated atmosphere; therefore, the grayscale values of the atmosphere were used as a reference for calculating the contrast index values in this experiment.

## Results

### (1) Contrast Increase

Under the employed scanning conditions, samples could not be detected, unless infiltrated with potassium permanganate, Lugós solution, phosphotungstate, lead citrate, bismuth tatrate, or uranium acetate. Infiltration with iron diamine, aqueous iodine, and alcoholic iodine did not allow detection of the samples.

Contrast improvement varied significantly among contrasting agents and among tissues. Table 3 lists the four most efficient contrasting agents for each tissue studied and the corresponding maximal mean contrast index (CI).

**Table 3:** Ranking of four best contrasting agents per tissue with best infiltration time.

Tissue	Best agent	Med. Max CI	2^nd^ best agent	Med. Max CI	3^rd^ best agent	Med best CI	4^th^ best agent	Med. Max CI
Sepal	OsO4 2d	0.94	Bi 8d	0.92	W/EtOH 0.5d	0.56	W/FAA 2d	0.5
Petal	Bi 8d	0.47	Pb 1d	0.44	Mn 2d	0.13	OsO4 2d	0.13
Filament	Bi 8d	0.6	Pb 1d	0.25	OsFeCN 8d	0.22	W/EtOH 0.5d	0.16
Pollen	OsO4 2d	2.55	W/FAA 2d	1.78	OsFeCN 8d	1.63	W/EtOH 0.5d	1.58
Stigma	OsO4 1d	1.23	Pb 0.5d	0.48	OsFeCN 8d	0.37	W/EtOH 2d	0.34
Ovary wall	W/FAA 2d	0.92	W/EtOH 2d	0.76	Pb 1d	0.57	OsFeCN 8d	0.54
Ovules	W/EtOH 0.5d	1.2	W/FAA 2d	1.17	Bi 8d	0.92	OsO4 2d	0.56

Footnote: Abbreviations: Bi = bismuth tartrate; Mn = potassium permanganate; OsO4 = osmium tetroxide; OsFeCN = osmium tetroxide with ferrocyanate; Pb = lead citrate; W/EtOH = phosphotungstate in 70% EtOH; W/FAA = phosphotungstate in FAA.

**Figure 2:**
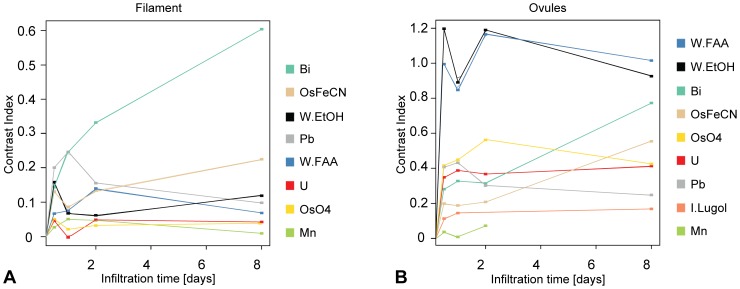
Contrast improvement over time for different floral tissues. (A) Contrast index *vs.* time for all contrasting agents on stamen filament. Filament cells are highly vacuolated, and the highest contrast index values are obtained with bismuth tartrate and osmium tetroxide and lead citrate, all of which bind strongly to the cell wall components. (B) Contrast index *vs.* time for all contrasting agents on ovules. Ovule cells are highly cytoplasmic, the highest contrast index values are obtained with phosphotungstate, that binds proteins and cell membranes. Abbreviations: Bi = bismuth tartrate; I.lugol = Lugol’s solution; Mn = potassium permanganate; OsO4 = osmium tetroxide; OsFeCN = osmium tetroxide with ferrocyanate; Pb = lead citrate; U = uranyl acetate; W/EtOH = phosphotungstate in 70% EtOH; W/FAA = phosphotungstate in FAA. Samples infiltrated with alcoholic and aqueous iodine are not detectable under the used scanning conditions.

Solvent-referenced CI values typically range between 0 and 2.5. The lower CI values were measured for highly vacuolated tissues, *e.g.* filaments (Fig. 2A), or petals (Fig. S2B), and the higher values were measured for the most cytoplasm- and protein-rich tissues, *e.g.* ovules (see Fig. 2B) or pollen (Fig. S2C). Bismuth tartrate, osmium tetroxide, and lead citrate displayed the highest contrast increase on the more vacuolated tissues (petals, sepals, and filaments; see Fig. 2A, S2A, and S2B). Phosphotungstate (in ethanol or in FAA), osmium tetroxide (with or without ferrocyanate), lead citrate, and bismuth tartrate displayed the highest contrast increase on the more cytoplasm- and protein-rich tissues (ovules, ovary wall, and pollen; see Fig. 2B, S2C, and S2E). CI values typically increased with time; however, for some contrasting agents (especially phosphotungstate), CI values after two days were higher than after 8 days (Fig. 2B).

### (2) Infiltration Speed, Reproducibility

**Figure 3:**
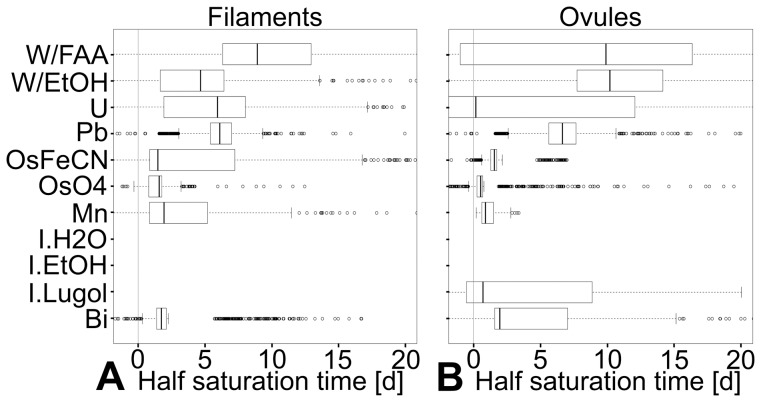
Reproducibility and speed of contrast improvement: distribution of half-saturation of contrast agents per tissue. The mathematical model for the saturation of infiltration agents (C(t) = C_∞_*e^(−1/kt)^ ) is regressed on 1000 random permutations of data points (one per time point). The coefficients obtained *via* these regressions allow the calculation of 1000 half-saturation times by applying the equation: 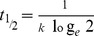 (A) Half-saturation times distribution for stamen filaments. (B) Half-saturation times distribution for ovules. In both vacuolated and cytoplasmic tissues, the fastest and most consistent (narrow spread) reagents are the most reactive ones: permanganate and osmium tetroxide (both strong oxidants), and bismuth tartrate (which is in 2N sodium hydroxide). Although making for the highest contrast increase in cytoplasmic tissues, phosphotungstate appears relatively slow (due to high saturation values) and comparatively little reliable (large spread of half-saturation values). Abbreviations: Bi = bismuth tartrate; I.lugol = Lugol’s solution; Mn = potassium permanganate; OsO4 = osmium tetroxide; OsFeCN = osmium tetroxide with ferrocyanate; Pb = lead citrate; U = uranyl acetate; W/EtOH = phosphotungstate in 70% EtOH; W/FAA = phosphotungstate in FAA. Samples infiltrated with alcoholic and aqueous iodine are not detectable under the used scanning conditions.

Different tissues absorb different contrasting agents at different speeds. For osmium tetroxide, the median half-saturation time, which was used as a proxy for saturation speed, was 0.5d in ovules, but 1.5d in filaments (Fig. 3A and 3B). However, some general trends could be observed: the contrasting agents for which the infiltration speed was the fastest (shortest median half-saturation time) and most constant (narrowest half-saturation time distribution) were osmium tetroxide, followed by bismuth tartrate, osmium tetroxide with ferrocyanate, and permanganate (Fig. 3 and S3). Lead citrate and uranyl acetate ranked next in median half-saturation time. Although allowing for the largest contrast increase, phosphotungstate (in EtOH or in FAA) ranks consistently as one of the slowest (longest half-saturation time) and least constant contrasting agents (broadest distribution of half-saturation time; see Fig. 3 and S3).

**Figure 4:**
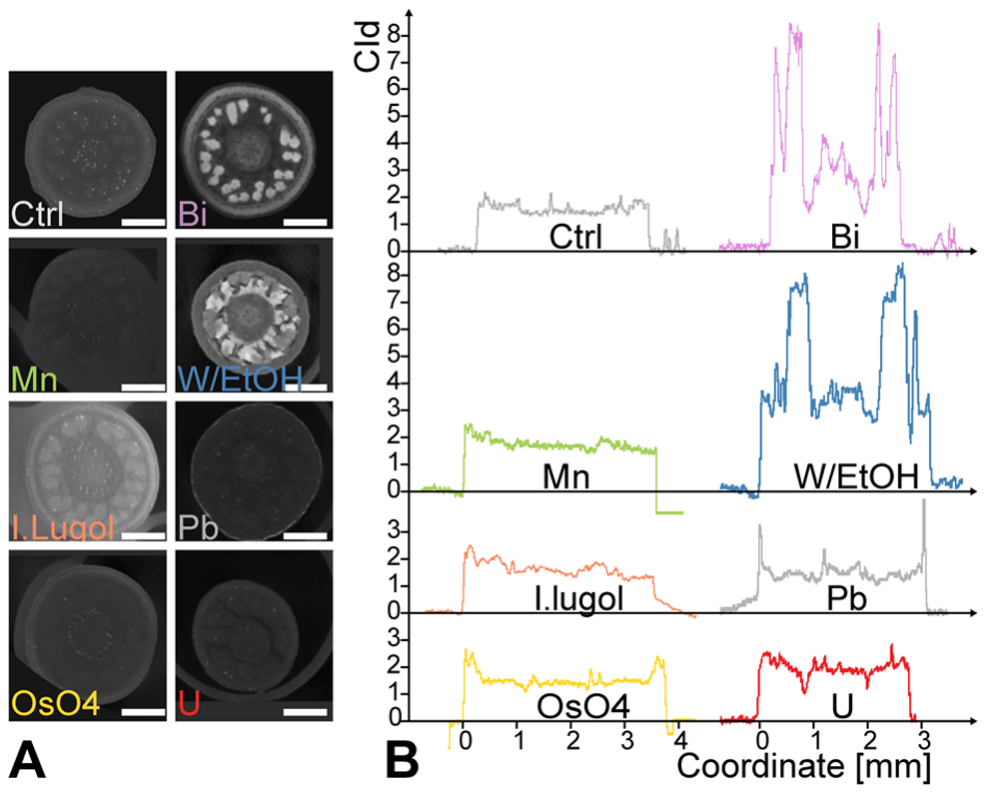
Tissue penetration: different contrasting agents penetrate the thick buds of *Marcgravia caudata* with different efficiencies. (A) Reconstructed transverse sections through buds of *M. caudata* after 8 days infiltration with selected contrast agents. The sections were chosen at the level of the developing stigma (center) and thecae. Note the thick protective calyptra (fused petals). Scale bar = 1 mm. (B) Air-referenced contrast index profiles through the pictures from part (A). Bismuth tartrate and phosphotungstate clearly outperform other stains for contrast increase and sample penetration. The lead carbonate crystals deposited on the surface of the buds induce two sharp peaks. Osmium tetroxide fails to penetrate thick samples as evidenced by higher contrast index values on the outer portion of the buds, but lower levels on the inside of the bud. Abbreviations: Bi = bismuth tartrate; I.lugol = Lugol’s solution; Mn = potassium permanganate; OsO4 = osmium tetroxide; OsFeCN = osmium tetroxide with ferrocyanate; Pb = lead citrate; U = uranyl acetate; W/EtOH = phosphotungstate in 70% EtOH; W/FAA = phosphotungstate in FAA.

On *Marcgravia caudata* flower buds, only a subset of the contrasting agents were qualitatively tested (see Fig. 4). Phosphotungstate and bismuth tartrate displayed increases in CI values at least two times higher than all the other contrasting agents tested (Fig. 4A, B). Phosphotungstate and bismuth tartrate also allowed for the largest differences of CI values among the tissues of the same sample (Fig. 4B). Uranyl acetate, potassium permanganate and Lugol’s iodine appear to have penetrated the sample (Fig. 4B) but did not cause strong increase in CI values. Osmium tetroxide did not penetrate the sample well, causing a strong increase in the CI values of the outer layer of the sample only (Fig. 4A, 4B). Lead citrate did not penetrate through the sample well and formed X-ray absorbing precipitates on the sample periphery (Fig. 4A, B).

### (3) Selectivity

**Figure 5:**
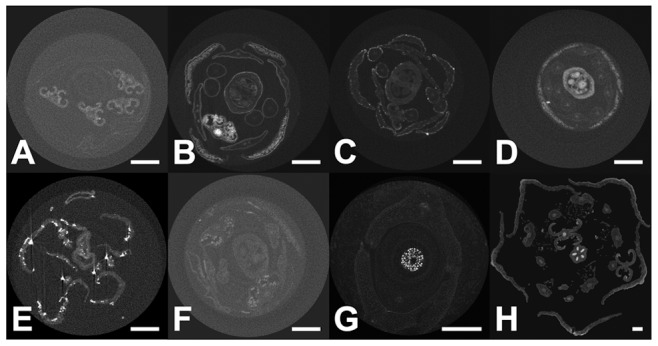
Reproducibility and uniformity, sample damage, and specificity of contrasting agents. (A) Permanganate typically contrasts one part of the sample and not the rest: *A. thaliana* flower after 2d infiltration with well contrasted thecae, and barely visible gynoecium. (B) Osmium tetroxide does not penetrate well inside samples and tends to accumulate on the periphery of organs (*A. thaliana*, 8d infiltration). (C) Lead citrate precipitates in presence of CO2 and forms lead carbonate crystals that accumulate at the sample periphery. The crystals absorb large amounts of X-rays, and cause reconstruction artifacts. (D) *A. thaliana* flower contrasted with phosphotungstate (8d), showing near ideal properties: good sample penetration and differential contrast increase. (E) and (F) *A. thaliana* flower, sample degradation after 2d in bismuth tartrate, and 2d in permanganate, respectively. (G) Specific staining of the nucellus of *Calycanthus floridus* by bismuth tartrate (8d) infiltration (possibly due to the presence of starch). (H) Specific staining of the PTTT of *Haplophyllum lissonotum* by phosphotungstate (possibly due to the presence of glycoproteins).

Our results indicate that all the used contrasting agents exhibit some level of specificity, as most tissues differed in their CI values (see Fig. 2, S2, and 4B). The scans were performed with a voxel size of 1.5 µm, which does not allow to visualize cellular details. Therefore, the observed “tissue specificity” appears to be mostly related to tissue vacuolization (with the exception of pollen). A specific contrasting agent for a given tissue was not found *per se*; however, very strong CI values for specific tissues were obtained in some species. Phosphotungstate, for example, contrasted the pollen tube transmitting tract of *Haplophyllum lissonotum* 6.5 times more than the surrounding tissues in the flower (Fig. 5H). Bismuth tartrate increased the CI value of the nucellus of *Calycanthus floridus* 30 times more than the surrounding tissues in the carpel (Fig. 5G). The selective infiltration of the pollen tube transmitting tract could be due to the high affinity of phosphotungstate for glycoprotein in en-bloc infiltration [38]. The selective infiltration of the nucellus of *C. floridus* by bismuth could be due to the presence of starch, to which bismuth has been documented to bind with high specificity [38].

### (4) Material Integrity

X-ray scanning is non-destructive at the morphological level; however, some infiltration protocols caused detectable sample damage. Sample damage was observed after only 1d infiltration in bismuth tartrate (Fig. 5E). The solution of bismuth tartrate contains 2N of sodium hydroxide, which rendered the samples very delicate and easy to damage. Infiltration with permanganate caused visible damage to samples as soon as after 2d infiltration (Fig. 5F), and infiltration for 8 days usually resulted in total sample loss. Infiltration with Lugoĺs solution causes visible sample damage after several weeks of infiltration.

## Discussion

**Figure 6:**
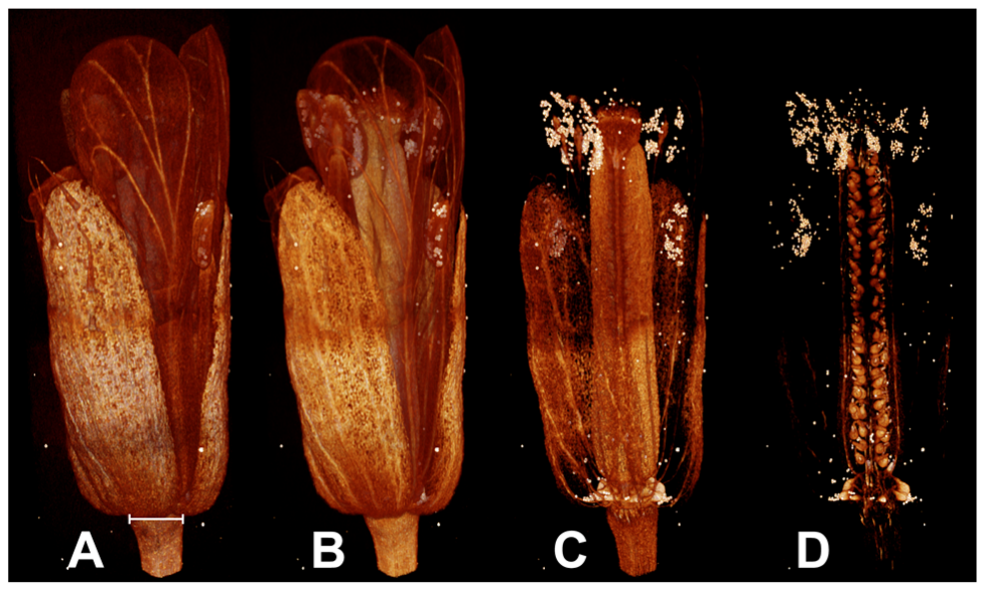
Virtual dissection of *A. thaliana* flower: differential contrast increase allows to handle different floral parts independently. (A–D) Identical 3D model of the same *A. thaliana* flower visualized with narrower and narrower selection of pixel grayscale (in D, only the brightest pixels are displayed). A, Full model with broadest selection of pixel grayscale. (B) From the petals, only the vasculature remains. (C),Petals and filaments, and parts of the sepals are removed; the ovary and the pollen grains remain. (D) Most of the flower is removed, and only the ovules, their vasculature and the pollen grains remain. Diameter of peduncke highlighted in (A) = 372 µm.

Our protocols allow for strong contrast increase on all the tested material. Moreover, contrasting flowers with phosphotungstate leads to contrast increase two to five times greater than with iodine or with uranium acetate (Fig. 2 and S2); therefore, our protocols clearly outperform previously published protocols [36], [37]. Not only does phosphotungstate increase contrast significantly better than any other contrasting agent, but it also penetrates even thick and dense tissues well (Fig. 4). Finally, the differences in contrast increase between tissues leads to different grayscales on the scan data, which in turn allow to virtually dissect these tissues from each other *via* grayscale thresholding (Fig. 6).

### (1) Contrast Increase

Differences in contrast increase between tissues is best explained by the nature of the contrasting agents: relatively small, charged molecules that bind relatively unspecifically to charged surfaces (membranes), charged solubilized molecules (*e.g.* proteins, DNA), and Lewis bases (*e.g.* DNA bases).

The contrast index (CI) values after two days infiltration which were higher than after 8 days could be due to increased leaching of the contrasting agent from the sample into the solvent. An increase of the grayscale of the solvent would lead to a decrease of the CI values (see equation (1)). Leaching of phosphotungstate is furthermore well-documented [38]. Similar irregularities within infiltration times in the 0-5-2d range could be caused by small differences in the 70% EtOH washing steps of the samples prior to mounting.

### (2) Speed and Reproducibility

In *Arabidopsis*, the contrasting agents with the fastest (shortest half-saturation time) and most constant speed (narrowest half-saturation time distribution) are the following: osmium tetroxide, bismuth tartrate, osmium tetroxide with ferrocyanate, and permanganate. All are comparatively reactive contrasting agents: osmium tetroxide and permanganate are relatively strong oxidants and bismuth tartrate is in an extremely basic 2N sodium hydroxide solution. The long median infiltration times observed for phosphotungstate could be due to three factors: (1) leaching from the sample into the mounting solvent for long infiltration times, (2) leaching from the sample during sample washing, and (3) the fact that saturation concentrations of phosphotungstate can be very high (prolonged exposure to phosphotungstate has been observed to allow up to a doubling of the dry weight of some samples [38]). The broad half-saturation time distributions observed for infiltration with phosphotungstate could be due to the same causes as those that appear to be responsible for long median infiltration times (leaching of the contrasting agent in the solvent and irregularities in washing steps).

In larger samples, such as *Marcgravia caudata* buds, penetration of the sample is the limiting factor for contrast increase. Only phosphotungstate and bismuth tartrate perform well. Osmium tetroxide, on the contrary, fails to penetrate the samples. The poor penetration of osmium tetroxide for en-bloc infiltration is a well described phenomenon [38].

### (3) Uniformity

Most contrasting agents increased the contrast of the samples uniformly. However, potassium permanganate occasionally increased the contrast of only a part of the sample, leaving other parts unchanged (Fig. 5A), which makes it poorly suited for standard protocols. Osmium tetroxide often contrasts the external part of a sample much more heavily than its internal part, especially in large samples, *e.g.*, *Marcgravia* buds (Fig. 4B). Lead citrate precipitates in presence of carbon dioxide in the form of lead carbonate crystals [38] that accumulate on the surface of the sample. The crystals absorb very large amounts of X-Rays (Fig. 5C), thereby causing reconstruction artefacts, which in turn leads to the production of a non-uniform 3D model (compare to phosphotungstate Fig. 5D).

### (4) Small Samples Protocol

**Figure 7:**
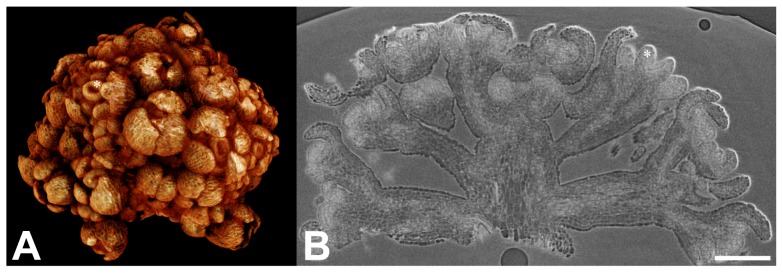
Special protocol for very small and soft objects. *A. thaliana* flower meristems of double line *ap1 cal pAP1::AP1-GR* mutant (courtesy Toshiro Ito research group, Temasek Life Sciences Laboratory, National University of Singapore, unpublished data) 6d after induction of flower development. Flowers were infiltrated 1 week in phosphotungstate in FAA, critical point dried, sputtered with gold and mounted in glue. (A) 3D model. (B) Reconstructed longitudinal section of the inflorescence. The cells of the meristem are resolved. Asterisk denotes a developing gynoecium. Scale bar = 100 µm.

For very small samples (<1 mm), which are usually scanned at high resolution, movements of the sample during the scan are an acute problem. This can be further amplified if the sample has a low intrinsic X-ray absorbance, as is the case for most non lignified tissues (*e.g.* meristems). For such samples, it is recommended to infiltrate 1–8d in phosphotungstate, to dehydrate by drying (critical point), to sputter coat with gold, and to mount in a drop of glue directly on a metal stub. Such a protocol allows the visualization with unprecedented resolution of small, soft plant samples with a commercial HRXCT system (Fig. 7A, 7B; see also Video S1 and Video S2). This protocol allows imaging with a maximal sample resolution of 3 µm (cell walls in Fig. 7B). This resolution is very close to the maximal resolution of our equipment (1.5 µm) and allows to resolve even meristematic cells (Fig. 7B).

### (5) Reduction of Shrinking and Leaching Artefacts

One of the most powerful aspects of computed tomography is the production of quantitative 3D data, which can be correlated with other datasets. If plant material is most commonly fixed in FAA, it is usually stored in 70% EtOH. During storage in 70% EtOH, plant material tends to shrink; however, FAA causes little shrinkage of the material [38]. Since phosphotungstate is soluble in FAA, an “all in FAA protocol” is possible, *i.e.*, fixation, storage, infiltration (with phosphotungstate), and scanning, in FAA. When precise quantitative datasets are aimed for (*e.g.*, pollination studies), such a procedure can be used to minimize shrinking artefacts.

Leaching of phosphotungstate out of the sample can be prevented by scanning the sample in the same solution as it was infiltrated in. However, leaching of phosphotungstate out the sample could also be turned into an advantage, because it could allow removal of most of the heavy metal from the sample after scanning by washing the sample with solvent. Loss of contrast up to the level of untreated samples was observed, but complete removal of phosphotungstate from a sample was not tested. This could allow to borrow and to return, intact, rare samples from spirit collections.

### (6) Virtual Dissection *via* Grayscale Thresholding and Applications (Pollen Count)

Selective contrast increase *via* phosphotungstate infiltration allows grayscale thresholding of the scan data (selection of a bracket of grayscales to be displayed). The latter does not only allow to separate the sample from the background, but also to separate the different parts of the sample. Therefore, it is possible, to some extent, to virtually dissect a well infiltrated sample by choosing different selections of grayscales to be displayed, *e.g.* gynoecium, ovules and vasculature (Fig. 6A–6D), or cell nuclei (*e.g.* nuclei of stigmatic papillae in *A. thaliana*; Fig. S4, Video S3 and S4). Medical Computed Tomography routinely employs a similar procedure to separate tissues (*e.g.* bone, muscle and fat) *via* tables of values of linearly transformed grayscale values called Hounsfield units [40]. Grayscale thresholding also allows to access 3D data of a sample subset, such as volumes, which in turn allow quantitative measurements. The pollen in the *Arabidopsis* flower in Fig. 6D can be separated from the rest of the sample, and the total number of pollen grains can be straightforwardly calculated with a small error, after the average volume of a pollen grain has been determined. The best volumetric estimation obtained was 880 grains, whereas a manual count yielded 900, *i.e.* a 2.2% error (see Fig. S5). Vasculature, ovules, or cell nuclei can also be studied in the same way. Our protocols therefore open up the way for *in silico* phenotyping [41], [42] of inner organs and cell nuclei.

### (7) Conclusion

Overall, the best performing contrasting agents, which do not visibly damage the samples are the contrasting agents comprising phosphotungstate (in EtOH or in FAA) and osmium tetroxide (with or without ferrocyanide). Given the penetration limitations of osmium, it is suggested to restrict its use to open and thin material (open buds, tissues only a few cells thick). Furthermore, phosphotungstate (in EtOH or in FAA) and osmium tetroxide (with or without ferrocyanide) clearly outperform previously described infiltration agents based on iodine [36] and on uranium [37]. For routine use, phosphotungstate is recommended, because it is efficient both in increasing contrast and in penetrating even thick samples. In addition, it is little toxic.

Although our results allow the straightforward use of CT for qualitative morphological work [43], quantitative applications are now more straightforward as well. Our results allow for the streamlined acquisition of high-resolution phenotypic data, which can be correlated with genomic, transcriptomic, or metabolomic data *via* the setting of morphometric landmarks, in order to advance our understanding of phenotypic evolution and developmental trajectories [1]. Morphological fits between flowers and pollinators can also be quantified. Plants shape change and cell elongation can also be straightforwardly quantified. Finally, due to the preferential binding of phosphotungstate to meristematic tissues, floral development can be studied quantitatively (length and volume information in mutant *vs.* ctrl) in plants in which the young reproductive organs are difficult to access (inside stems or covered by many bracts). *In silico* phenotyping of plant inner parts, *e.g*., ovules, vasculature, pollen, and cell nuclei, is now possible *via* grayscale thresholding.

## Supporting Information

Figure S1 **Literature statistics: percentage of paper containing “computed tomography” per Subject Area** ***vs.*** **time.**
(TIF)

Figure S2 **Contrast improvement over time for different floral tissues.**
(TIF)

Figure S3 **Reproducibility and speed: distribution of the half-saturation times estimated by permutations of data points.**
(TIF)

Figure S4 **Grayscale thresholding on** ***A. thaliana*** **(wt ecotype Col-0) stigma reveal germinating pollen and cell nuclei.**
(TIF)

Figure S5 **Total pollen grain number estimates from volumetric assessment after thresholding.**
(TIF)

Table S1 **Literature statistics: comparative underuse of computed tomography in plant sciences.**
(DOCX)

Video S1 **Longitudinal section series played as a video though the inflorescence in** **Fig. 7** **.**
(MPG)

Video S2 **Animation of a 3D model of the inflorescence in** **Fig. 7** **(*A. thaliana* flower meristems of double line *ap1 cal pAP1::AP1-GR* mutant; courtesy Toshiro Ito research group, Temasek Life Sciences Laboratory, National University of Singapore, unpublished data), virtually cut at the level of the section in** **Fig. 7** **.**
(MPG)

Video S3 **Stigma of** ***Arabidopsis thaliana*** **(wt ecotype Col-0) with grayscale thresholding set to remove solvent signal.**
(MPG)

Video S4 **Stigma of** ***Arabidopsis thaliana*** **(wt ecotype Col-0). Grayscale thresholding set to remove most of papillae.**
(MPG)

## References

[1] Bellaire A, Ischebeck T, Weinhäuser I, Staedler YM, Schönenberger J, et al. (2012) The developmental trajectory of *Arabidopsis* flowers. 23rd International Conference on Arabidopsis Research. Vienna. pp. 178.

[2] van der Niet T, Zollikofer CPE, de Leon MSP, Johnson SD, Linder HP (2010) Three-dimensional geometric morphometrics for studying floral shape variation. Trends in Plant Science 15: 423–426. 20541450 10.1016/j.tplants.2010.05.005

[3] Jiang N, Yang WN, Duan LF, Xu XC, Huang CL, et al (2012) Acceleration of CT reconstruction for wheat tiller inspection based on adaptive minimum enclosing rectangle. Computers and Electronics in Agriculture 85: 123–133.

[4] Weninger WJ, Geyer SH, Mohun TJ, Rasskin-Gutman D, Matsui T, et al (2006) High-resolution episcopic microscopy: a rapid technique for high detailed 3D analysis of gene activity in the context of tissue architecture and morphology. Anatomy and Embryology 211: 213–221. 16429276 10.1007/s00429-005-0073-x

[5] Goebel JC, Bolbos R, Pinzano A, Schaeffer M, Rengle A, et al (2008) In vivo rat knee cartilage volume measurement using quantitative high resolution MRI (7 T): feasibility and reproducibility. Biomed Mater Eng 18: 247–252. 19065030

[6] Rengle A, Armenean M, Bolbos R, Goebel JC, Pinzano-Watrin A, et al (2009) A dedicated two-channel phased-array receiver coil for high-resolution MRI of the rat knee cartilage at 7 T. IEEE Trans Biomed Eng. 56: 2891–2897. 10.1109/TBME.2008.2006015 19932985

[7] Schneider JE, Bamforth SD, Grieve SM, Clarke K, Bhattacharya S, et al (2003) High-resolution, high-throughput magnetic paragraph sign resonance imaging of mouse embryonic paragraph sign anatomy using a fast gradient-echo sequence. MAGMA 16: 43–51. 12695885 10.1007/s10334-003-0002-z

[8] Quintana L, Sharpe J (2011) Optical projection tomography of vertebrate embryo development. Cold Spring Harb Protoc 2011: 586–594. 21632785 10.1101/pdb.top116

[9] Sharpe J (2009) Optical Projection Tomography. In: Sensen CW, Hallgrímsson B, editors. Advanced Imaging in Biology and Medicine: Technology, Software Environments, Applications. Berlin: Springer. pp. 199–220.

[10] Hounsfield GN (1980) Computed medical imaging. Science 210: 22–28. 6997993 10.1126/science.6997993

[11] Hounsfield GN (1973) Computerized transverse axial scanning (tomography). 1. Description of system. Br J Radiol 46: 1016–1022. 4757352 10.1259/0007-1285-46-552-1016

[12] Hounsfield GN (1976) Historical notes on computerized axial tomography. J Can Assoc Radiol 27: 135–142. 789383

[13] Gregory PJ, Hutchison DJ, Read DB, Jenneson PM, Gilboy WB, et al (2003) Non-invasive imaging of roots with high resolution X-ray micro-tomography. Plant and Soil 255: 351–359.

[14] Hamza MA, Anderson SH, Aylmore LAG (2001) Studies of soil water drawdowns by single radish roots at decreasing soil water content using computer-assisted tomography. Australian Journal of Soil Research 39: 1387–1396.

[15] Heeraman DA, Hopmans JW, Clausnitzer V (1997) Three dimensional imaging of plant roots in situ with x-ray computed tomography. Plant and Soil 189: 167–179.

[16] Kaestner A, Schneebeli M, Graf F (2006) Visualizing three-dimensional root networks using computed tomography. Geoderma 136: 459–469.

[17] Perret JS, Al-Belushi ME, Deadman M (2007) Non-destructive visualization and quantification of roots using computed tomography. Soil Biology & Biochemistry 39: 391–399.

[18] Pierret A, Capowiez Y, Moran CJ, Kretzschmar A (1999) X-ray computed tomography to quantify tree rooting spatial distributions. Geoderma 90: 307–326.

[19] Tracy SR, Roberts JA, Black CR, McNeill A, Davidson R, et al (2010) The X-factor: visualizing undisturbed root architecture in soils using X-ray computed tomography. Journal of Experimental Botany 61: 311–313. 20051353 10.1093/jxb/erp386

[20] DeVore ML, Kenrick P, Pigg KB, Ketcham RA (2006) Utility of high resolution x-ray computed tomography (HRXCT) for paleobotanical studies: An example using London Clay fruits and seeds. American Journal of Botany 93: 1848–1851. 21642129 10.3732/ajb.93.12.1848

[21] Friis EM, Crane PR, Pedersen KR, Bengtson S, Donoghue PCJ, et al (2007) Phase-contrast x-ray microtomography links cretaceous seeds with Gnetales and Bennettitales. Nature 450: 549–U511. 18033296 10.1038/nature06278

[22] von Balthazar M, Pedersen KR, Crane PR, Stampanoni M, Friis EM (2007) Potomacanthus lobatus gen. et sp nov., a new flower of probable Lauraceae from the Early Cretaceous (Early to Middle Albian) of eastern North America. American Journal of Botany 94: 2041–2053. 21636397 10.3732/ajb.94.12.2041

[23] Pika-Biolzi M, Hochuli PA, Flisch A (2000) Industrial X-ray computed tomography applied to paleobotanical research. Rivista Italiana Di Paleontologia E Stratigrafia 106: 369–377.

[24] Tafforeau P, Boistel R, Boller E, Bravin A, Brunet M, et al (2006) Applications of X-ray synchrotron microtomography for non-destructive 3D studies of paleontological specimens. Applied Physics a-Materials Science & Processing 83: 195–202.

[25] Cloetens P, Mache R, Schlenker M, Lerbs-Mache S (2006) Quantitative phase tomography of Arabidopsis seeds reveals intercellular void network. Proceedings of the National Academy of Sciences of the United States of America 103: 14626–14630. 16973748 10.1073/pnas.0603490103 PMC1600010

[26] Yamauchi D, Tamaoki D, Hayami M, Uesugi K, Takeuchi A, et al (2012) Extracting Tissue and Cell Outlines of Arabidopsis Seeds using Refraction Contrast X-Ray CT at the SPring-8 Facility. International Workshop on X-Ray and Neutron Phase Imaging with Gratings 1466: 237–242.

[27] Matsushima U, Hilger A, Graf W, Zabler S, Manke I, et al (2012) Calcium oxalate crystal distribution in rose peduncles: Non-invasive analysis by synchrotron X-ray micro-tomography. Postharvest Biology and Technology 72: 27–34.

[28] Milien M, Renault-Spilmont AS, Cookson SJ, Sarrazin A, Verdeil JL (2012) Visualization of the 3D structure of the graft union of grapevine using X-ray tomography. Scientia Horticulturae 144: 130–140.

[29] Korte N, Porembski S (2011) Anatomical Analysis of Turgescent and Semi-Dry Resurrection Plants: The Effect of Sample Preparation on the Sample, Resolution, and Image Quality of X-Ray Micro-Computed Tomography (mu CT). Microscopy Research and Technique 74: 364–369. 20734408 10.1002/jemt.20917

[30] Korte N, Porembski S (2012) A morpho-anatomical characterisation of Myrothamnus moschatus (Myrothamnaceae) under the aspect of desiccation tolerance. Plant Biology 14: 537–541. 22300275 10.1111/j.1438-8677.2011.00546.x

[31] Leroux O, Knox JP, Masschaele B, Bagniewska-Zadworna A, Marcus SE, et al (2011) An extensin-rich matrix lines the carinal canals in Equisetum ramosissimum, which may function as water-conducting channels. Annals of Botany 108: 307–319. 21752793 10.1093/aob/mcr161 PMC3143055

[32] Brodersen CR, Lee EF, Choat B, Jansen S, Phillips RJ, et al (2011) Automated analysis of three-dimensional xylem networks using high-resolution computed tomography. New Phytologist 191: 1168–1179. 21569032 10.1111/j.1469-8137.2011.03754.x

[33] Herman GT (2009) Fundamentals of Computerized Tomography: Image Reconstruction from Projections, Second Edition. Berlin: Springer. 300 p.

[34] Metscher BD (2009) MicroCT for Developmental Biology: A Versatile Tool for High-Contrast 3D Imaging at Histological Resolutions. Developmental Dynamics 238: 632–640. 19235724 10.1002/dvdy.21857

[35] Metscher BD (2009) MicroCT for comparative morphology: simple staining methods allow high-contrast 3D imaging of diverse non-mineralized animal tissues. BMC Physiol 9: 11. 19545439 10.1186/1472-6793-9-11 PMC2717911

[36] Dhondt S, Vanhaeren H, Van Loo D, Cnudde V, Inze D (2010) Plant structure visualization by high-resolution X-ray computed tomography. Trends in Plant Science 15: 419–422. 20542721 10.1016/j.tplants.2010.05.002

[37] Leroux O, Leroux F, Bellefroid E, Claeys M, Couvreur M, et al (2009) A new preparation method to study fresh plant structures with X-ray computed tomography. Journal of Microscopy-Oxford 233: 1–4. 10.1111/j.1365-2818.2008.03088.x 19196405

[38] Hayat M (2000) Principles and techniques of electron microscopy. Cambridge: Cambridge University Press.

[39] Jain AK (1989) Fundamentals of digital images processing. Englewood Cliffs, NJ: Prentice Hall International.

[40] Brooks RA (1977) A quantitative theory of the Hounsfield unit and its application to dual energy scanning. J Comput Assist Tomogr 1: 487–493. 615229 10.1097/00004728-197710000-00016

[41] Kaminuma E, Hasegawa Y, Heida N, Yoshizumi T, Nakazawa M, et al (2005) Three-dimensional shape modeling and in silico phenotypic analysis of Arabidopsis by using the micro X-ray computed tomography. Plant and Cell Physiology 46: S62–S62.

[42] Kaminuma E, Yoshizumi T, Wada T, Matsui M, Toyoda T (2008) Quantitative analysis of heterogeneous spatial distribution of Arabidopsis leaf trichomes using micro X-ray computed tomography. Plant Journal 56: 470–482. 18643999 10.1111/j.1365-313X.2008.03609.x

[43] Gamisch A, Staedler YM, Schönenberger J, Fischer GA, Comes HP (2013) Histological and Micro-CT Evidence of Stigmatic Rostellum Receptivity Promoting Auto-Pollination in the Madagascan Orchid *Bulbophyllum bicoloratum* . PLoS ONE 8: e72688. 23967332 10.1371/journal.pone.0072688 PMC3742538

