# Supplementary figures and images for: Plant Tissues in 3D via X-Ray Tomography: Simple Contrasting Methods Allow High Resolution Imaging
Source: PLoS One. 2013 Sep 27;8(9):e75295. doi: 10.1371/journal.pone.0075295 (PMC3785515; doi:10.1371/journal.pone.0075295)

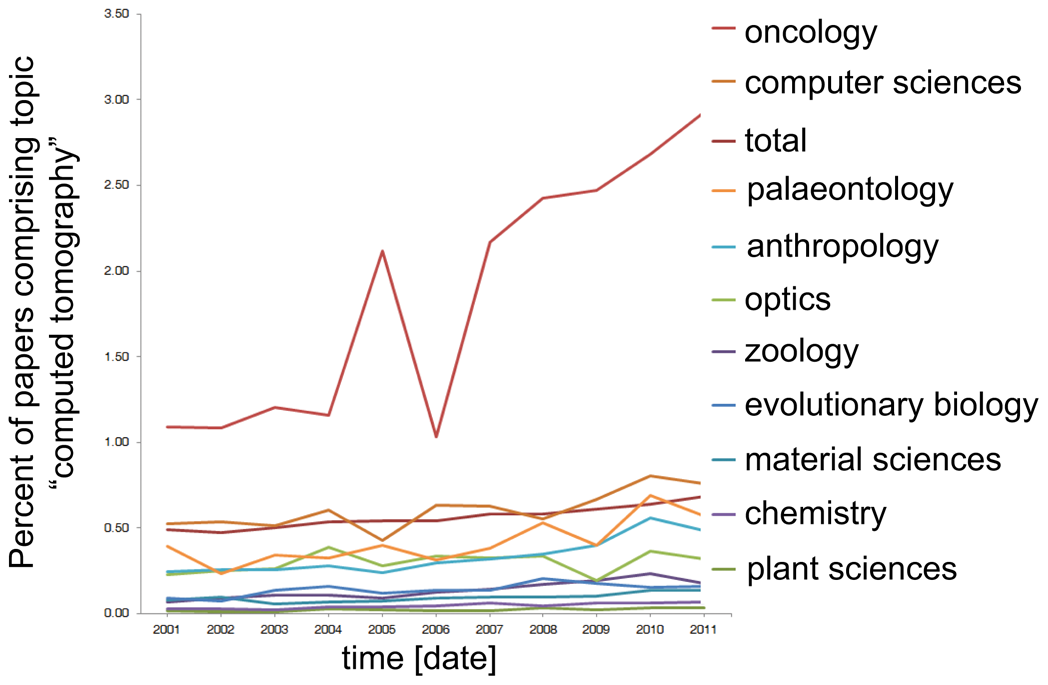

Supplement: Figure S1 — Literature statistics: percentage of paper containing “computed tomography” per Subject Area vs. time. (TIF) [file pone.0075295.s001.tif]

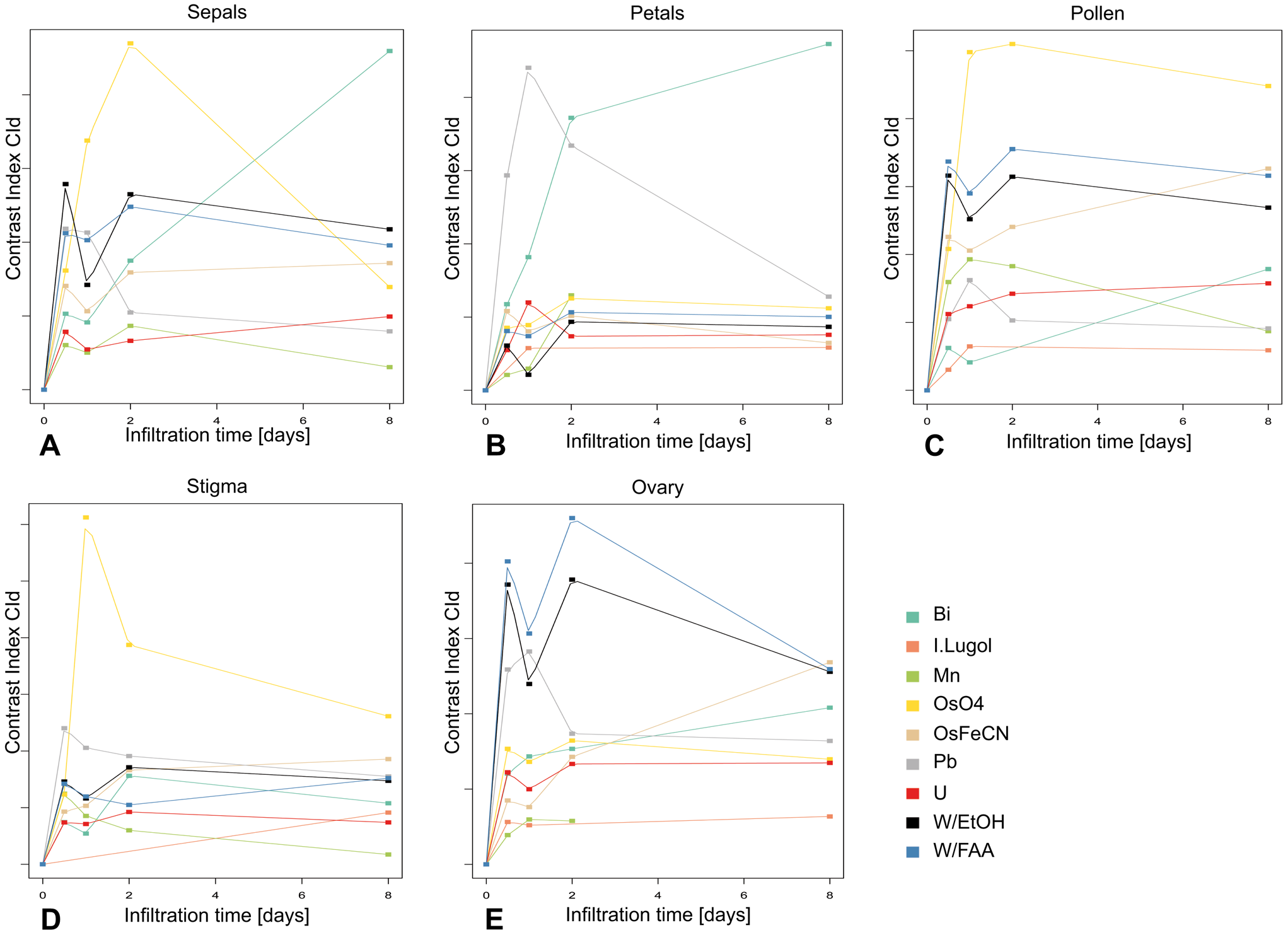

Supplement: Figure S2 — Contrast improvement over time for different floral tissues. (TIF) [file pone.0075295.s002.tif]

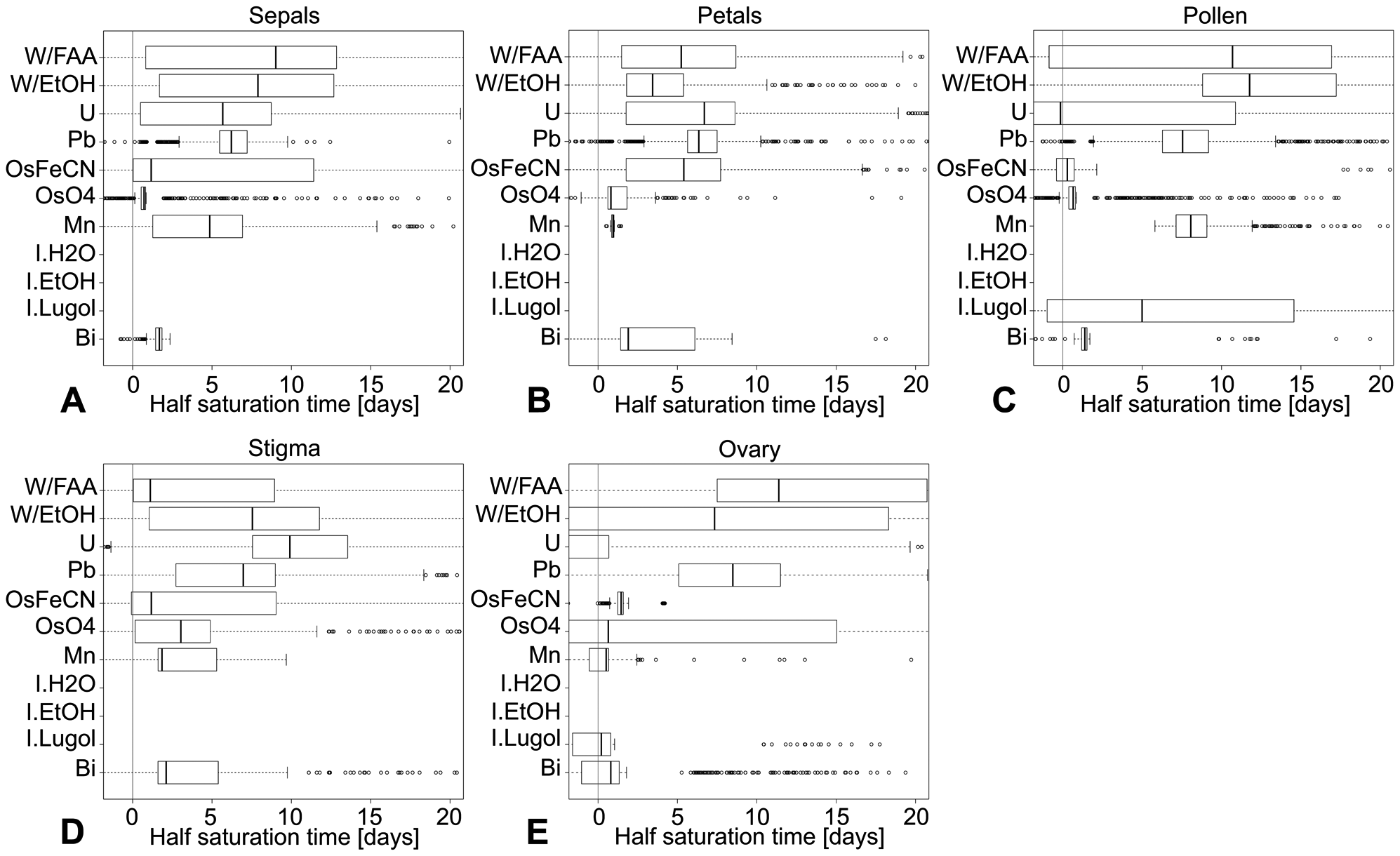

Supplement: Figure S3 — Reproducibility and speed: distribution of the half-saturation times estimated by permutations of data points. (TIF) [file pone.0075295.s003.tif]

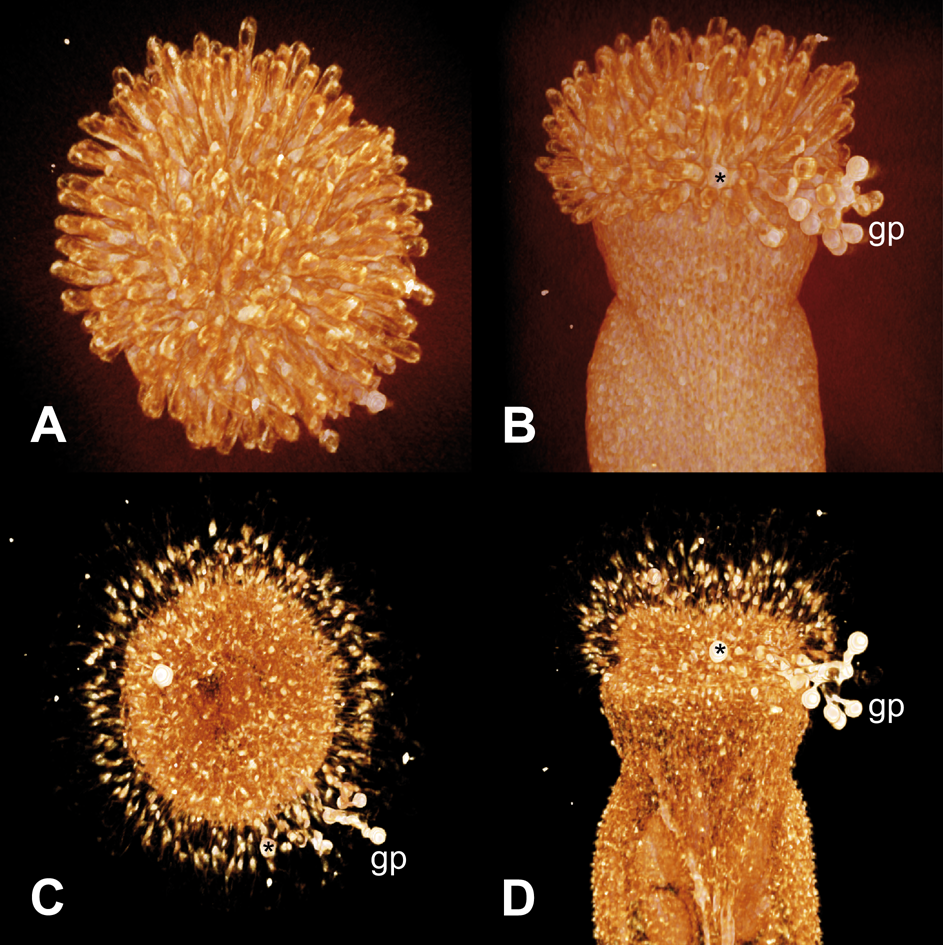

Supplement: Figure S4 — Grayscale thresholding on A. thaliana (wt ecotype Col-0) stigma reveal germinating pollen and cell nuclei. (TIF) [file pone.0075295.s004.tif]

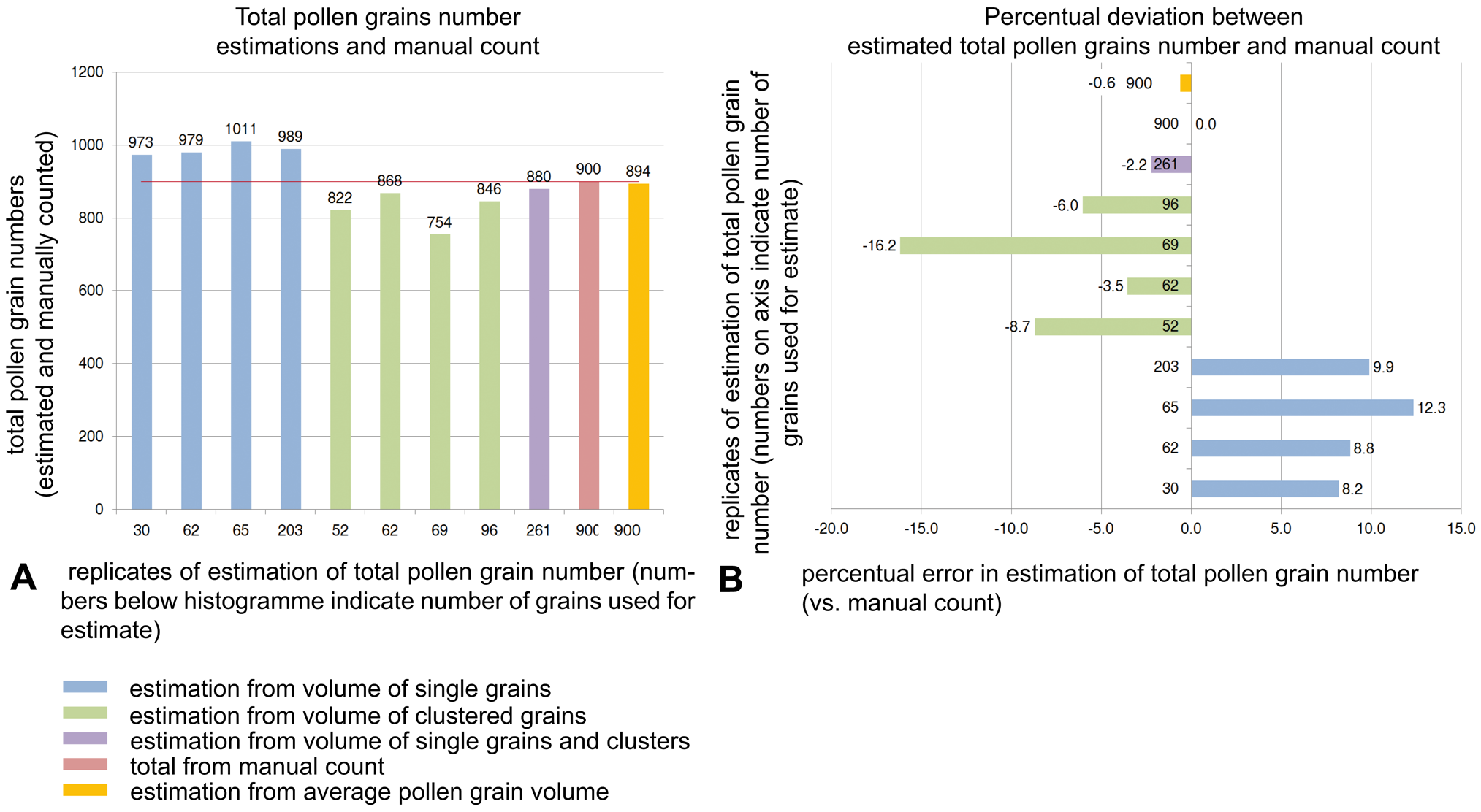

Supplement: Figure S5 — Total pollen grain number estimates from volumetric assessment after thresholding. (TIF) [file pone.0075295.s005.tif]
