# Supplementary material for: Plant Tissues in 3D via X-Ray Tomography: Simple Contrasting Methods Allow High Resolution Imaging
Source: PLoS One. 2013 Sep 27;8(9):e75295. doi: 10.1371/journal.pone.0075295 (PMC3785515; doi:10.1371/journal.pone.0075295)
Supplement: Table S1 — Literature statistics: comparative underuse of computed tomography in plant sciences. (DOCX) [file pone.0075295.s006.docx]

**Table S1**

Literature statistics: comparative underuse of computed tomography in plant sciences

| Subject Area | Av. hit/y | Av. hit CT/y | Av. % hit CT | Slope %CT *vs.* t | R^2^ | p |
| --- | --- | --- | --- | --- | --- | --- |
| Oncology | 255775 | 4960 | 1.85 | 0.20 | 0.8 | 2×10^-4^ |
| Comp. sci. | 309991 | 1917 | 0.61 | 0.025 | 0.57 | 7.2×10^-3^ |
| Paleontology | 10786 | 46 | 0.42 | 0.030 | 0.56 | 8×10^-3^ |
| Anthropology | 19467 | 68 | 0.34 | 0.028 | 0.76 | 4.4×10^-4^ |
| Robotics | 23568 | 38 | 0.15 | 0.017 | 0.89 | 1.4×10^-5^ |
| Zoology | 63398 | 91 | 0.14 | 0.014 | 0.85 | 6×10^-5^ |
| Evol. biol. | 19633 | 28 | 0.14 | 0.008 | 0.51 | 1.3×10^-2^ |
| Mat. sci. | 243717 | 238 | 0.09 | 0.006 | 0.59 | 5.5×10^-3^ |
| Chemistry | 346140 | 170 | 0.05 | 0.004 | 0.91 | 4.7×10^-6^ |
| Agriculture | 111607 | 29 | 0.03 | 0.002 | 0.56 | 7.8×10^-3^ |
| Env. sci. ecol. | 206329 | 54 | 0.02 | 0.003 | 0.45 | 2.4×10^-2^ |
| **Plant sciences** | **63195** | **14** | **0.02** | **0.002** | **0.50** | **1.5**×**10^-2^** |
| All areas | 6309428 | 35977 | 0.56 | 0.019 | 0.95 | 4.4×10^-7^ |

Footnote: The Subject Areas are a journal-based classification by ISI Web of Knowledge^SM^. Searches were performed with the year alone, and then were refined by Research Area to obtain hits per year (averaged over 10 years in second column). The same searches with the year and the topic “Computed tomography” were used to obtain the results averaged in the 3^rd^ column. The 4^th^ to 7^th^ column display statistics over the years 2000-2011. av. = average; y = year; CT = Computed Tomography; t = time.
